# Panobinostat in combination with bortezomib and dexamethasone in multiply relapsed and refractory myeloma; UK routine care cohort

**DOI:** 10.1371/journal.pone.0270854

**Published:** 2022-07-07

**Authors:** Nadjoua Maouche, Bhuvan Kishore, Zara Bhatti, Supratik Basu, Farheen Karim, Sharadha Sundararaman, Freya Collings, Bing Tseu, Heather Leary, Noel Ryman, Udaya Reddy, Grant D. Vallance, Jaimal Kothari, Karthik Ramasamy

**Affiliations:** 1 Department of Haematology, Oxford University Hospitals NHS Foundation Trust, Oxford, United Kingdom; 2 Department of Haematology, University Hospitals Birmingham NHS Foundation Trust, Birmingham, United Kingdom; 3 Department of Haematology, The Royal Wolverhampton NHS Trust, Wolverhampton, United Kingdom; 4 Department of Haematology, Great Western Hospitals NHS Foundation Trust, Swindon, United Kingdom; 5 Department of Haematology, Buckinghamshire Healthcare NHS Trust, Bucks, United Kingdom; 6 Department of Haematology, Milton Keynes University Hospital NHS Foundation Trust, Milton Keynes, United Kingdom; 7 Department of Haematology, Hampshire Hospitals NHS Foundation Trust, Basingstoke, United Kingdom; Bari University Aldo Moro, ITALY

## Abstract

The combination of panobinostat, bortezomib and dexamethasone (PanBorDex) is available as a treatment option for relapsed refractory multiple myeloma (RRMM) based on the PANORAMA-1 trial which investigated this triplet in early relapse. In routine clinical care, PanBorDex is used primarily in later relapses and is commonly administered in attenuated dosing schedules to mitigate the treatment-related toxicity. We set out to evaluate efficacy and safety outcomes with PanBorDex later in the disease course and evaluate the role of attenuated dosing schedules. This was a retrospective evaluation of patients treated in routine clinical practice between 2016–2019 across seven heamatology centres in the UK; patients who received at least one dose of PanBorDex were eligible for inclusion. The dosing schedule of panobinostat (10mg, 15mg or 20mg, twice or three times a week) and bortezomib (0.7mg/m^2^, 1mg/m^2^ or 1.3mg/m^2^ once or twice weekly) was as per treating physician choice. Patients received treatment until disease progression or unacceptable toxicity. The primary outcome is response rates according to IMWG criteria. Key secondary endpoints include progression-free survival (PFS) and overall survival (OS). Other secondary endpoints include rates of adverse events according to CTCAE criteria. In total, 61 patients were eligible for inclusion and received PanBorDex primarily as ≥5^th^ line of treatment. One third of patients received PanBorDex at full dose, for the remaining two thirds, treatment was given in reduced dose intensities. The overall response rate was 44.2%, including 14.7% very good partial response (VGPR) rates; 68.8% of patients derived clinical benefit with stable disease or better. The median PFS was 3.4 months; non-refractory patients and those who achieved VGPR benefited from prolonged PFS of 11.4 months and 17.7 months, respectively. The median OS was 9.5 months. The triplet was associated with 45% and 18% incidence of grade 3–4 thrombocytopenia and diarrhea, respectively.

## Introduction

The therapeutic landscape for relapsed/refractory multiple myeloma (RRMM) has evolved considerably with the approval of several novel agents [1]. There is no clear consensus on the optimal combination and sequence of treatment in early and later relapses [2]. The decision making process is influenced by individual patient- and disease-related factors such as frailty, comorbidities, genetic risk status, but mostly by prior exposure; depth, duration of responses and drug resistance patterns with previous therapies [3].

In earlier relapses, the goal of treatment is to achieve deep responses and durable disease-free intervals [4]. Current treatment options include highly effective immunomodulatory agent (IMiD)- or proteasome inhibitor (PI)-based doublet or triplet regimen incorporating lenalidomide, bortezomib, the next generation PIs carfilzomib and ixazomib, the third generation IMiD pomalidomide and the anti-CD38 monoclonal antibody daratumumab, with many patients receiving ongoing continuous therapy and progressing on treatment limiting these options in later relapses [5].

In the multiply relapsed setting, patients are likely to present with more aggressive disease that is resistant to multiple therapeutic options [6], leading to poor responses, shorter remissions and unfavorable outcomes [7]. In this setting of advanced disease, the goal of treatment is to achieve disease control [5]. One treatment strategy is to initiate a new class agent to which the patient is naïve to target resistant clones [4]. Re-treatment with an agent previously used in a fixed duration and to which the patient responded, like bortezomib, may also be considered [8]. The combination of panobinostat, a potent pan-histone deacetylase inhibitor (HDACi), with bortezomib and dexamethasone (PanBorDex) showed synergism via dual targeting of the aggresome and proteasome pathways and offers an alternative treatment option [9]. It is approved for RRMM based on the PANORAMA1 trial which compared PanBorDex vs. Placebo-BorDex in patients with relapsed myeloma who were not refractory to bortezomib [10]. Subgroup analysis demonstrated greatest benefit amongst patients who received ≥ 2 prior therapies that included bortezomib and an IMiD; progression-free survival of 12.5months in the panobinostat arm versus 4.7months for placebo, leading to its approved indication in this setting [11]. Despite clinical benefit, the combination was associated with significant toxicity burden particularly high rates of grade 3–4 haematological and gastrointestinal adverse events making treatment delivery challenging [10].

In our practice, PanBorDex is used as a later-line treatment option (≥ 5th line) where patients often present with increasing frailty, cumulative symptom and toxicity burden with a significant impact on quality of life (QoL) [12]. As a result, the regimen is delivered in reduced doses and attenuated schedules to improve tolerability and preserve QoL. There is very little published experience from routine clinical care on the role of PanBorDex in re-capturing responses in a multiply relapsed, heavily pretreated, refractory setting. There are very few reports from routine practice investigating attenuated dosing schedules with this regimen to inform how to optimally deliver it within the challenges of the real-world setting.

## Material and methods

### Study design and data collection

We conducted a multicentre, retrospective review of multiply relapsed myeloma patients treated with PanBorDex in routine clinical care between January 2016 and June 2019 across seven haematology centres in the UK.

At the physician’s discretion, patients received Panobinostat (PAN) doses 10mg, 15mg or 20mg twice or three times a week, plus Bortezomib (BTZ) 0.7mg/m^2^, 1mg/m^2^ or 1.3mg/m^2^ subcutaneously once or twice weekly and dexamethasone. Treatment was delivered for two weeks in 21-day cycles until disease progression or unacceptable toxicity. All patients who received at least one dose of treatment were eligible for inclusion.

Data was retrospectively collected from patients’ medical records including baseline demographics and disease characteristics; age, sex, time of diagnosis, Charlson Co-morbidity index (CCI), International Staging System (ISS) and high-risk disease features. Treatment data was pulled from chemotherapy databases including previous anti-myeloma treatment exposure and refractoriness, PAN and BTZ dosing and treatment duration. Data was entered anonymously onto an electronic case report form. The study was approved locally by the Clinical Governance Committee.

### Study endpoints

Outcomes evaluated included treatment response rates [overall response rate (ORR), complete response (CR), very good partial response (VGPR), partial response (PR), stable disease (SD) and clinical benefit rate (CBR≥SD)] assessed according to International Myeloma Working Group (IMWG) response criteria [13], progression-free survival (PFS) and overall survival (OS). Subgroup analysis for PFS was performed based on depth of response, disease refractoriness to PIs and IMiDs. We have also explored the impact of using varying dosing strategies on ORR and PFS.

PFS was defined as the time between treatment initiation and disease progression (as defined by IMWG response criteria) or death. OS was evaluated as the time from the start of treatment to death from any cause.

Safety evaluation included; the rate and grading of adverse events (AEs) according to Common Terminology Criteria for Adverse Events, version 4.0 [14], and frequencies of dose reductions and treatment discontinuation due to AEs.

### Statistical analyses

Data were analyzed using IMB® SPSS software package (version 26.0). Descriptive statistics for quantitative variables are presented as median (interquartile range [IQR] or range) and as number and percentage (%) for categorical variables. Kaplan-Meier method was used for PFS and OS estimates and presented as median (95% confidence intervals [95% CI]) with log-rank test for subgroup comparisons.

## Results

### Baseline cohort characteristics

In total, 61 patients treated with PanBorDex and were included in this evaluation. Details on patient demographics, disease features and prior treatment data are described in Table 1.

**Table 1:** Cohort’s baseline demographics, disease characteristics and previous treatment data.

(Total N = 61)	
**Age** Median (range)- Years	72 (43–85)
**Sex- no (%)** Male Female	35 (57.6) 26 (42.6)
**Time since diagnosis (years)** Median	5.6
**ISS Staging- no (%)** ISS I ISS II ISS III Data unavailable	7 (11.5) 16 (26.2) 29 (47.5) 9 (14.8)
**Cytogenetic Features no (%)** Standard Risk High Risk Data Unavailable	4 (6.5) 20 (32.7) 37 (60.6)
**Charlson Comorbidity Index (CCI)** ≤ 3 ≥4 **Pre-existing cardiovascular comorbidity- n (%)**	23 (37.7) 37 (60.6) 18 (29.5)
**Disease Category–no (%)** Relapsed Refractory Primary Refractory	18 (29.5) 42(68.9) 1 (1.6)
**Prior lines of therapies** Median (range)	4 (1–7)
**Prior PI- no (%)** Any PI* Bortezomib Ixazomib Carfilzomib **PI-refractory**^&^	60 (98.3) 58 (95) 15 (24.5) 8 (13.1) 29 (47.5)
**Prior IMiDs- no (%)** Any IMiD Thalidomide Lenalidomide Pomalidomide **IMiD- Refractory**^&&^	61 (100) 48 (78.6) 56 (91.8) 21 (34.4) 50 (83.3)
**Double refractory to IMiD and PI- no (%)**	27 (44.3)
**Prior Daratumumab- no (%)**	11 (18)
**Prior HSCT- no (%)**	24 (39.3)
Other prior therapies: 2 vorinostat (2 patients), bendamustine (4 patients), melflulen (2 patients)	

*17 patients (27.8%) received 2 prior PI-based therapies, and 6 patients (9.8%) received 3 prior PI-based therapies.

^&^ Patients could be refractory to ≥ 1 PI: 27.9%, 24.6% and 8.2% were refractory to bortezomib, ixazomib and carfilzomib, respectively.

^&&^ Patients could be refractory to ≥ 1 IMiD; 18%, 65.6% and 32.8% were refractory to thalidomide, lenalidomide, and pomalidomide respectively.

Abbreviations: ISS, International Staging System for multiple myeloma; PI, Proteasome Inhibitor; IMiD, Immunomodulatory agent; ASCT, Autologous stem cell transplantation.

This real-world cohort includes a largely elderly and comorbid population with a median age of 72 years, and 60% of patients had a CCI ≥ 4. At baseline, 70% of patients had disease refractory to their most recent therapy. One third of patients (32.7%) were known to have high-risk disease defined as presence of one or more of the adverse risk cytogenetic abnormalities (CAs); t(4;14), t(14;16), t(14;20), del(17p), and/or gain(1q).

In this multiply relapsed cohort, patients had a median of four prior lines of treatment. Most patients (95%) received previous bortezomib-based treatment. Second generation PIs, ixazomib and carfilzomib, were previously given in 24.5% and 13.1% of patients, respectively. All patients received previous treatment with an IMiD including pomalidomide in 34.4% of patients. Almost half of the cohort (47.5%) was PI-refractory, most patients (83.3%) were IMiD-refractory, in total 44.3% were double refractory. Other prior therapies included daratumumab (11 patients), vorinostat (2 patients), melflufen (2 patients).

### PanBorDex dosing and exposure

At treatment initiation, one third (32.8%) of patients received PanBorDex dosing in line with the PANORAMA-1 schedule i.e., PAN 20mg three times a week (TW) plus BTZ 1.3mg/m^2^ twice (BW) weekly. For the remaining two thirds (67.2%) of patients, treatment was started at attenuated doses and/or reduced frequency of either PAN and/or BTZ. In total, 60.6% of patients received BTZ once weekly and 57.3% had PAN dosed at 20mg TW. Details on PAN and BTZ dosing schedules are summarized in Table 2.

**Table 2:** Panobinostat and bortezomib dosing schedules at treatment initiation.

N = 61			
Bortezomib dose and frequency	n (%)	Panobinostat dose and frequency	n (%)
1.3mg/m^2^ TWICE weekly*	22 (36)	20mg Three times weekly^&^	35 (57.3)
1.3mg/m^2^ ONCE weekly**	33 (54.1)	20mg TWICE weekly^&&^	10 (16.4)
1mg/m^2^ TWICE weekly*	2 (3.2)	10mg Three times weekly^&^	10 (16.4)
1mg/m^2^ ONCE weekly**	4 (6.5)	Other^$^	6 (9.8)

*Cycles 1–8: days 1, 4, 8 and 11, from Cycle 9: days 1 and 8 only.

** On days 1, 8 and 15.

^&^On days 1, 3, 5, 8, 10 and 12.

^&&^On days 1, 5, 8 and 12.

^$^Other dosing schedules for panobinostat; 20mg ONCE weekly (4 patients), 15mg TWICE weekly (1 patient), 10mg TWICE weekly (1 patient)

The median follow up duration for the cohort was 35.9 months. At data cut off, patients received a median of 4 cycles of PanBorDex (range: 1–20); one third (29.5%) of patients completed ≥8 cycles of treatment.

Patients were on treatment for a median duration of 3.0 months 95% CI (2.2–3.7). At last follow up, all patients had discontinued treatment. The most common reasons for treatment discontinuation were disease progression (59%) and adverse events (23%).

### Responses, progression-free and overall survival

All patients were evaluable for response. The overall response rate (ORR ≥PR) in the total cohort was 44.2% (27/61 patients). Best responses included, VGPR; 14.7% (9/61 patients), PR; 29.5% (18/61 patients), MR; 11.5% (7/61 patients) and SD; 13.1% (8/61 patients), 31.1% (19/61) had progressive disease. Overall, a CBR (≥SD) of 68.8% was achieved.

**Fig 1:**
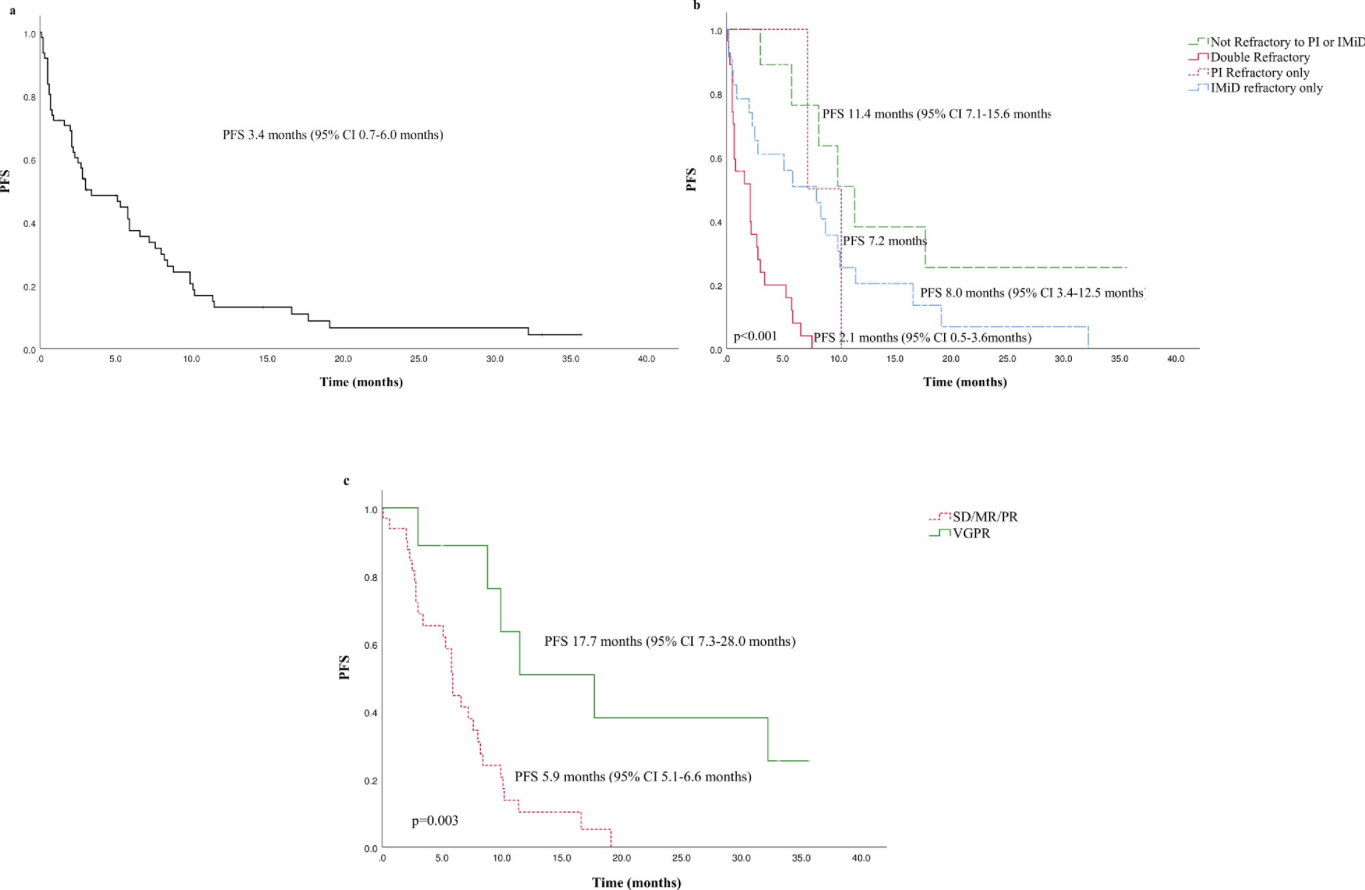
Kaplan-Meier estimates of progression free survival (PFS) for PanBorDex patients. (a) PFS of all patients in the cohort. (b) PFS according to refractoriness to PIs and IMiDs. (c) PFS according to depth of response. The PFS was significantly longer in non-refractory patients (*p*<0.001) and in patients who achieved a VGPR (*p* = 0.003). Abbreviations: PFS, progression-free survival; PI, proteasome inhibitor; IMiD, immunomodulatory agent; SD, stable disease; MR, minimal response; PR, partial response; VGPR, very good partial response.

The median progression-free survival (PFS) was 3.4months (95% CI 0.7–6.0 months), Fig 1A. 35 patients (57.3%) received a subsequent line of therapy; the median time to next treatment was 7.0 months (95% CI 4.9–9.0 months).

Longer progression-free survival was observed in non-PI/IMiD refractory patients and those who were IMiD-refractory only; 11.4 months and 8.0 months respectively, time to progression in double-refractory patients was 2.1 months, Fig 1B. Patients with at least stable disease had a PFS of 5.9 months, and those with VGPR achieved a significantly prolonged PFS of 17.7months (*p* = 0.003), Fig 1C. Patients with known high-risk CAs had a short PFS of 2.2 months. The median overall survival for the total cohort was 9.5 months (95% CI 5.0–14.9 months).

### ORR and PFS according to dosing

**Fig 2:**
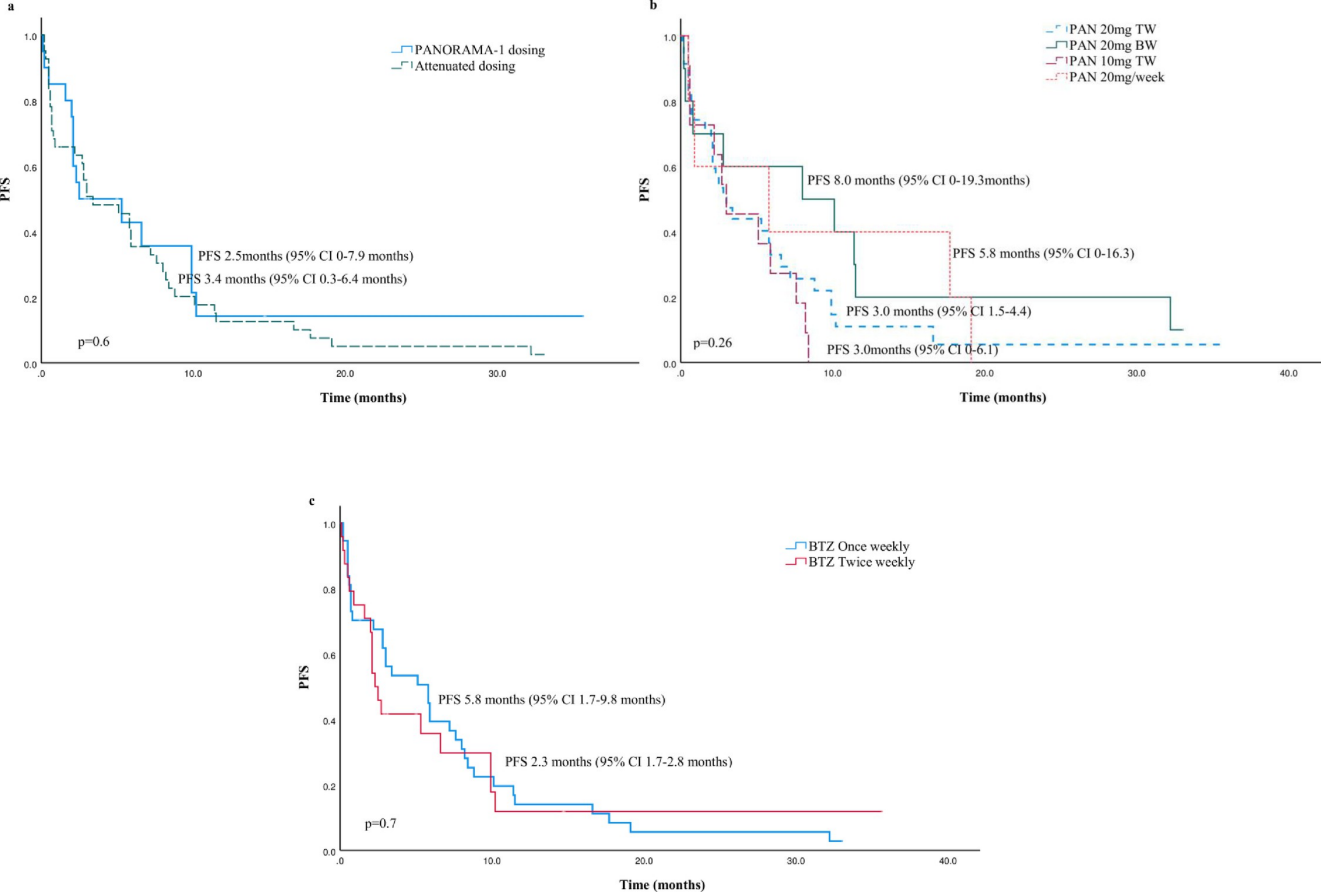
Kaplan-Meier estimates of progression free survival (PFS) for PanBorDex patients according to dosing. (a) PFS per PANORAMA-1 or attenuated dosing. (b) PFS according to PAN dosing schedules. (c) PFS according to BTZ dosing schedule. There was a trend for longer PFS in the PAN 20mg BW group and BTZ once weekly group. Abbreviations: PFS, progression-free survival; PAN, panobinostat; BTZ, bortezomib; TW, three times a week; BW; twice weekly.

Higher response rates were observed in patients who received PanBorDex in line with PANORAMA-1 trial dosing compared to those who received attenuated doses; ORR, 55% vs. 39% respectively, Table 3. However, there was no significant difference in PFS between the two groups, 2.5 months vs. 3.4 months respectively, *p* = 0.6, Fig 2A.

**Table 3:** Overall response rate according to dosing.

Characteristic	PanBorDex Dosing		PAN dosing				BTZ dosing	
	PANORAMA-1 dosing n = 20	Attenuated dosing n = 41	20mg TW n = 35	20mg BW n = 10	10mg TW n = 11*	20mg/week n = 5**	Twice weekly n = 24	Once weekly n = 37
ORR (%)	**55%**	**39%**	48.5%	50%	36.3%	20%**	50%	40.5%
Median age (yrs)	**67.5**	**73**	69	76	70	75	68.5	73
High risk CA, n (%)	**6 (30)**	**14 (34)**	10 (28.5)	2 (20)	4 (36.3)	4 (80)	9 (37.5)	11 (29.7)
PI refractory, n (%)	**10 (50)**	**19 (46.3)**	19 (54.2)	2 (20)	6 (54.5)	2 (40)	13 (54.2)	16 (43.2)
IMiD refractory, n (%)	**16 (80)**	**34 (82.9)**	28 (80)	8 (80)	10 (90.9)	4 (80)	20 (83)	30 (81)
Double Refractory, n (%)	**9 (45)**	**18 (43.9)**	17 (48.5)	2 (20)	6 (54.5)	2 (40)	12 (50)	15 (40)

*Includes one patient who received 15mg twice a week.

** One patient with 1qgain achieved VGPR and received 12 cycles of treatment, another patient with del17p had SD and continued on treatment for 16 cycles.

Abbreviations: PanBorDex, Panobinostat in combination with bortezomib and dexamethasone; PAN, panobinostat; BTZ, bortezomib; TW, three times a week; BW; twice weekly; ORR, overall response rate; CA, cytogenetic abnormalities; PI, proteasome inhibitor; IMiD, immunomodulatory agent

A greater proportion of patients in the PAN 20mg TW and PAN 20mg BW groups achieved a response; 48.5%, and 50% respectively vs. ORR 36.3% in the PAN 10mg TW group, Table 3. The PFS was longer in the PAN 20mg BW group; 8.0 months vs. 3.0 months in the PAN 20mg TW and 10mg TW groups, (p = 0.2), Fig 2B, although there were less high-risk and PI-refractory patients in the PAN 20mg BW group. Among the 5 patients who received PAN 20mg/week, one patient with 1qgain achieved a VGPR and had a PFS of 17.7 months, another patient with del(17p) maintained SD for 19.1 months.

The ORR was higher in the BTZ twice weekly group vs. once weekly group; 50% vs. 40.5% respectively, Table 3. There was a trend towards longer time to progression in the BTZ once weekly compared BTZ twice weekly group; PFS 5.8months vs. 2.3months respectively (*p* = 0.7), Fig 2C, although the latter had a greater proportion of high-risk and PI-refractory patients.

### Adverse events evaluation

The most common adverse events (AEs) of special interest are presented in Table 4. Most patients (86.9%) had at least one treatment-related AE, grade 3–4 AEs occurred in 68.9% of patients. The most commonly observed grade 3–4 AEs were thrombocytopenia and diarrhea, reported in 45.9% and 18% of patients, respectively. Other AEs included infections (31.1%), fatigue (21.3%), peripheral neuropathy (19.7%) and pneumonia (11.4%). Twenty-five patients had baseline ECGs and repeat ECGs monitoring whilst on therapy; of those, 2 patients had an increase in QTcF prolongations (>60 msec) from baseline, both patients had pre-existing heart failure.

**Table 4:** Adverse event (AEs) of special interest observed during PanBorDex treatment, graded according to CTCAE 4.0 toxicity grading criteria.

AEs	All grades % (n)	Grade 3/4% (n)
**Any AE**	86.9 (53)	68.9(42)
**Thrombocytopenia**	52.5 (32)	45.9 (28)
**Neutropenia**	9.8 (6)	8.1 (5)
**Diarrhea**	36 (22)	18 (11)
**Peripheral Neuropathy**	19.7 (12)	6.5 (4)
**Fatigue**	21.3 (13)	9.8 (6)
**Nausea & Vomiting**	9.8 (6)	-
**Sepsis**	11.5 (7)	-
**Pneumonia**	11.4 (7)	-
**Infections**	31.1 (19)	-

The most commonly observed G3/4 toxicities were thrombocytopenia (45.9%) and diarrhea (18%). Other common AEs included; peripheral neuropathy (19.7%), fatigue (21.3%), and infections (31.1%)

Abbreviations: AEs, Adverse Events

Serious adverse events reported during therapy included arrhythmias (3 patients), heart failure (2 patients), liver impairment (3 patients), acute renal failure (6 patients). One patient developed venous thromboembolism and one patient was diagnosed with posterior reversible encephalopathy syndrome related to bortezomib. On-treatment death events occurred in 14 patients; 3 were infection-related deaths, the remaining patients died of progressive disease.

Treatment discontinuation due to AEs occurred in 23% of patients. Nearly half of the patients (46%) had at least one dose reduction to either panobinostat, bortezomib or both.

## Discussion

In this real-world cohort we report outcomes with PanBorDex when used in later relapses and when administered in varying mitigated dosing schedules. In this setting of advanced disease, a notable overall response rate of 44.2% was observed; the median time to progression was 3.4months which led to an overall survival of 9.5 months. In a group with limited treatment options, over two-thirds derived a clinical benefit and remained progression-free for a median of 5.9 months. These results suggest that this regimen could offer a treatment option to maintain a level of disease control in the presence of multiple clones later in the disease course, probably due to the varied anti-myeloma activity of panobinostat on several intracellular pathways [15]. Notably, in a small subset of patients, deep responses were obtained and led to a significantly prolonged PFS of 17.7months; these patients had previously achieved deep responses on prior exposure to fixed-duration bortezomib-based therapy, suggesting that PanBorDex could offer a suitable treatment option to recapture clinically meaningful responses in PI-exposed disease with PI-sensitive clones.

In a group predominantly lenalidomide-exposed and IMiD-refractory, patients benefitted from a progression-free interval of 8.0 months suggesting that this regimen may offers a possible treatment options in later relapses for patients progressing on lenalidomide therapy, especially that continuous therapy with lenalidomide-based combinations are becoming the mainstay of treatment in front-line and later relapses [16].

Overall, the progression-free survival obtained in our cohort is inferior to PFS of 12.5month reported in PANORAMA-1 trial. However, our population is very different; patients are more heavily pre-treated, pre-dominantly PI-exposed and refractory, whilst the PANORAMA-1 trial excluded PI-refractory patients. Altogether, these differences preclude comparing PanBorDex efficacy reported in the original trial to its true benefit within our routine practice treatment algorithm [17]. The outcomes observed in our cohort appeared similar to the ORR 34.5% and median PFS of 5.4 months reported in the PANORAMA-2 trial which evaluated PanBorDex in a more closely aligned population of heavily pre-treated and bortezomib-refractory patients with a median of four prior therapies [18].

In multiply relapsed myeloma, patients often present with poor marrow reserve, co-morbidities and increasing frailty, which further complicates treatment delivery in the clinic. In our routine care experience, this regimen was frequently given in attenuated doses and frequencies to minimize toxicity burden. A small group of patients received treatment as stipulated in the PANORAMA-1 trial and had higher overall response rate of 55%. It is intriguing that despite the poorer baseline prognostic factors, responses in this group were mostly similar to ORR 58.9% seen in the subgroup of patient with ≥ 2 prior therapies including BTZ and an IMiD in the PANORAMA-1 trial, which further supports the potential role of this combination in achieving responses in later relapses. Most patients received modified dosing schedules; typically, in older and frailer patients, bortezomib was given once weekly and panobinostat could be adjusted to 20mg BW or 10mg TW. Slightly fewer responses (39%) were observed with modified schedules, however, there was no significant difference in progression-free survival which remained modest in both groups; possibly due to clonal evolution and the presence of unfavorable cytogenetics and dominant resistant clones leading to diminished progression-free survival despite optimal responses [19, 20].

The frequency of responses appeared to be higher in the panobinostat 20mg three times weekly, 20mg twice weekly group and with twice weekly bortezomib; however, these results should be interpreted with caution due to limited patient numbers and different baseline characteristics within each group which might have contributed to differences in outcomes. This observation however remains consistent with the results of PANORAMA-3 trial which investigated three different regimens of panobinostat with reduced bortezomib dosing to once weekly [21]. In this trial, the ORR were highest in the 20mg three times weekly and 20mg twice weekly groups; 62.2% and 65.1% respectively, compared to 50.6% in 10mg three times a week group, although the 10mg three times a week schedule was best tolerated [21]. It is however important to note that PANORAMA- 3 trial was not powered to show these differences, patients were less heavily pre-treated and the trial excluded patients who were refractory to BTZ [21].

Another real-world study evaluated this regimen in a similar multiply relapsed (≥5^th^) setting using a different schedule of panobinostat 20mg three times a week on days 1, 3, 5, 15, 17, 19 and bortezomib given at 1.3mg/m^2^ once weekly on Days 1, 8, 15 and 22 [22]. In their study, despite a higher proportion of patients (> two-thirds) having high-risk CAs, outcomes were similar to our cohort with a reported ORR 47% and median PFS of 3.5 months [22]. One limitation to be noted in our cohort is that cytogenetic testing was not repeated in a great proportion of patients. The observed extended PFS of 17.7 and 19.1 months seen in two patients with 1qgain and del(17p) respectively, is noteworthy of the longer-lasting anti-myeloma control with this combination in these difficult to treat patients. Altogether, these observations may suggest that the epigenetic modulation of panobinostat could potentially confer benefit in patients with poor prognostic factors, although small patient numbers preclude firm conclusions [23].

The overall survival suggests that panobinostat may have a potential role as a later-line treatment option, particularly as other highly active and well tolerated monoclonal antibody-based regimen are used preferentially in earlier relapses limiting their uses as subsequent salvage treatments. Notably, the overall survival observed in our cohort compares to OS of 9.2 months reported in triple- and quad-refractory patients who were treated after progression on daratumumab- or isatuximab- based treatment [24].

Overall, the safety profile mirrored that reported in the clinical trial with thrombocytopenia, gastrointestinal side effects, peripheral neuropathy and fatigue being the most observed treatment-emergent toxicities. Generally, thrombocytopenia was reversible and not associated with increased bleeding events, although regular hospital visits for platelet transfusion support maybe required. Whilst treatment-related thrombocytopenia is a well-established adverse effect of Panobinostat, in this setting of progressive disease it is likely that plasma cell infiltration in bone marrow further contributes to thrombocytopenia by triggering thrombopoietin (TPO) production [25].

In this elderly and comorbid cohort, where cardiac monitoring was performed, there were no major clinically significant QTc prolongation events or other new emerging cardiac safety signals; low rates of ECG changes, heart failure and arrhythmias were observed generally in patients with pre-existing cardiac history and these patients would require careful close monitoring. Infectious complications requiring hospital admissions were common in this setting of heavily pre-treated patients, reported cases of infection-related death events occurred whilst on treatment.

Generally, lower rates of grade 3–4 events were observed in our cohort compared to trials, probably due to the use of attenuated dosing schedules and once weekly subcutaneous bortezomib which has shown an improved tolerability profile [26–28]. However, further dose reductions to manage toxicities were still required in around half of the patient and around one quarter of the cohort discontinued therapy due to intolerability.

In view of the toxicity profile, patients require frequent hospital visits for regular monitoring and institution of supportive care measures [29]. Altogether, the associated healthcare resource utilisation burden, the impact on patients’ QoL, and difficulty to maintain dose intensity for maximum clinical benefit may limit the widespread use of this triplet in practice. Despite modified dosing schedules, the treatment could still prove challenging to deliver for some patients. Therefore, careful patient selection based on frailty scores, comorbidities, and pre-treatment bone marrow reserves, are key considerations for integrating this therapy in practice [30].

The results from this study should be considered in the light of some limitations. In routine care setting, treatment dosing schedules were employed at physicians’ discretion, which may have introduced an element of selection bias based on patients’ performance status and disease burden, possibly leading to differences amongst patients within the different dosing groups. Furthermore, few patients in our cohort had previous exposure to daratumumab, second and third generation PIs, which are becoming mainstay treatment for both frontline and early relapse disease, therefore the role and true benefit of any myeloma treatment, including PanBorDex, is likely to be influenced by evolving shifts in the treatment paradigm.

In summary, our routine care experience shows that panobinostat in combination with bortezomib and dexamethasone can have a role in attaining responses later in the disease course in selected groups of patients, including when given in mitigated dosing schedules. Overall, time to progression remained short; although, non-refractory patients with disease that is sensitive to PIs were more likely to achieve and sustain meaningful clinical benefit. However, the regimen can still be associated with important toxicities; therefore, patient selection, individualized dosing approaches and careful monitoring are crucial. Any efforts to maximize clinical benefit should be carefully balanced against minimizing toxicity and maintaining QoL.
